# The effects of walking on frailty, cognitive function and quality of life among inactive older adults in Saudi Arabia: a study protocol of randomized control trial by comparing supervised group-based intervention and non-supervised individual-based intervention

**DOI:** 10.1186/s12877-023-04200-w

**Published:** 2023-09-27

**Authors:** Ming Yu Claudia Wong, Palash Karmakar, Mezna A. Almarzooqi, Ryan E. Rhodes, Chun-Qing Zhang, Kai-ling Ou, Duan Yanping, Pak Kwong Chung, Nouf A. Alghamdi

**Affiliations:** 1 Department of Health and Physical Education, The Education University of Hong Kong, Hong Kong, China; 2 Department of Sport, Physical Education and Health, Hong Kong Baptist University, Hong Kong, China; 3 Department of Community Health Sciences, College of Applied Medical Sciences, King Saud University, Riyadh, Kingdom of Saudi Arabia; 4 Leaders Development Institute, Ministry of Sport, Riyadh, Kingdom of Saudi Arabia; 5 School of Exercise Science, Physical & Health Education, University of Victoria, Victoria, Canada; 6 Department of Psychology, Sun Yat-sen University, Guangzhou, China

**Keywords:** Frailty, Walking, Older adults, Supervised-based, Group-based, Ageing, Saudi Arabia

## Abstract

**Background:**

Kingdom of Saudi Arabia (KSA) demographics are also changing with the increase in life expectancy in the country and the rise in the number of older Saudi Arabians. Saudi Arabia has a higher rate of physically inactive people, and most of them are between the ages of 55–64. Walking is one of the most prevalent forms of physical activity in Saudi Arabia and the study showed that most people prefer walking for recreational and health benefits. Therefore, the purpose of the proposed study is to compare the effects of supervised group-based walking and non-supervised individual-based walking interventions on frailty, cognitive function and quality of life among inactive older adults in Saudi Arabia.

**Method:**

This study will apply a three-group (2 intervention groups and 1 control group), double-blinded, randomized controlled trial (RCT) to examine the effect of different forms of walking interventions on Saudi Arabia older adults’ frailty, cognitive function and quality of life immediately after a 16-week intervention, as well as the residual effects 12 weeks after completion of the interventions.

**Discussion:**

This study aims to determine whether supervised group walking and non-supervised individual walking lead to different effects. Given the limited evidence in the literature regarding Saudi Arabia older adults’ physical fitness and health, it is worthwhile to explore the effect of walking, as well as the forms of walking on improving the overall physical fitness and psychological status of older adults in Saudi Arabia. The findings of the current study could also create awareness of the government and the general public in Saudi Arabia of the ageing problems and the effective ways of achieving better intervention results.

**Trial registration:**

The trial is registered at the ClinicalTrials.gov PRS (Trial ID: NCT05151575; Date of first posted: 12/07/2021).

## Background

### Worldwide ageing

According to the United Nations, people’s chronological age 60 or 65 years or more are generally defined as the older age [1]. Due to the increase expectancy of life in the past century, the prevalence of the worldwide ageing population is apparent [2]. The older aged population is considered as the most rapidly rising segment among all age groups throughout the world and it is projected that within the next few decades, this group of the population will be increased more than double [1, 3].

Almost each country of the world is now experiencing with the rapid growth of the ageing population. In 2019, the global number of population aged 65 years or above were about 703 million, which is projected to become about 1.5 billion in 2050. Moreover, population aged 65 years or above had increased from 6 to 9% between the year 1990 and 2019. That proportion of the population is also projected to increase further to 16% in the year 2050 when it is also expected that one in every six persons of the world will be found to be older adults. Similarly, in Asian countries, the percentages of older adults are also increasing at a rapid pace and have almost doubled from the year 1990 to 2019 [1].

## Problems with ageing

The ageing process is a natural and a multidimensional progression of human life characterized by the progressive decline in tissue and organ functions, leading to increase the risk of several diseases and mortality. It causes changes in physiological, psychological and pathological conditions as well as the social status of people [3, 4]. Ageing is associated with the loss of body function, weakness, different diseases and death. Due to ageing, most of the older adults lead a sedentary life and also, they are the most physically inactive group of a community [5]. Research report suggests that longer duration of inactivity or sedentary lifestyle can trigger the onset of overweight or obesity, diabetes, bone and cardiovascular disorders, and increase the likelihood of mortality. The study also indicates that higher level of inactive lifestyle is significantly associated with lower levels of health-related quality of life [6]. In case of older adults, cognitive functions which include emotion, attention, periodic memory and others are also declined due to ageing [7, 8]. Additionally, frailty, a clinical syndrome that includes frequent chance of falling, physical disability, and delirium, which is the most common among the older adults [9]. Beside general chronic health complexities, older adults are also at higher risk of psychological health problems, mainly the depression [10] and feel loneliness along with inadequate social support [3, 11]. Previous study findings confirm that chronic diseases, social support and leisure activities are extensively associated with all the domains of quality of life for older adults [12]. Overall, older adults are the most disadvantaged group of the population who are more physically, psychologically, and economically vulnerable than the young adults and the average healthcare cost is almost double than that of other age groups [6].

### Ageing in Saudi Arabia

Like other countries of the world, the demographic trend is also changing in the Kingdom of Saudi Arabia (KSA) with the increase of life expectancy and number of Saudi Arabian older adults are increasing continuously [13, 14]. In 2013, the World Health Organization (WHO) estimated that around 4.3% of the population in the KSA were between the age range of 55 and 64 years. It was also predicted that Saudi Arabian population aged 65 years and above will continue to increase to make up to 18.4% of the total population in the year 2050 [15]. In Saudi Arabia, rate of physically inactive people is higher, and the study indicated that 66.6% of Saudi population was physically inactive and majority of them belonged to the age group of 55–64 years [16]. The older adults in Saudi Arabia are suffering from several age-related chronic diseases and among these, hypertension and diabetes mellitus are more prevalent. The other age-related health complications are stroke, dementia, osteoporosis, heart diseases, obesity, asthma and kidney problems [17]. The study revealed that due to bone and joint pain and sedentary life (69%), Saudi older adults’ participation in physical activity is lower and these factors influence the risk of developing disability and fall. Moreover, researchers also found that sleep problem and other chronic diseases were also associated with an increased risk of falls which leads to poorer quality of life among older adults in Saudi Arabia. As Saudi Arabian older adults are increasing rapidly, this population group is creating numerous challenges to the health care system, especially to the persons related to the health services [13].

### Physical activity for healthy ageing

To minimize age related health consequences among the older adults, physical activity which can be defined as any kind of bodily movement exerted by the skeletal muscles with the expenditure of energy or calorie, is considered as one of the most vital and effective form of lifestyle intervention [5, 18]. Typically, different form of activities like games and sports, household tasks, occupation related works, leisure activities and active transportation are included as the several forms of physical activities [18]. Regular practice of light or moderate intensity physical activities has been well documented as significantly effective in delaying the reduction of functional ability among the older adults and helps to maintain healthy ageing status [10]. Additionally, physical activity is predominantly effective for preventing different age-related diseases and the frequent incidence of fall and maintaining the independence and overall quality of life. Among different form of physical activities, walking is recognized as one of the most accepted and commonly recommended form of activities, especially effective for middle-aged as well as older adults who are unable to perform other forms of rigorous activities of recommended level (≥ 150 min of moderate intensity per week) [19]. Interestingly, walking is one of the most predominant forms of physical activity in Saudi Arabia and the study revealed that the majority of the people prefer walking for their recreation and health benefits purposes [20, 21].

### Benefits of group-based physical activity

Apart from forms of physical activity, the style of engaging in the activity might also affect the effects of the engagement. Given that physical activities can be performed either in group-based or individual-based, the previous study reported that group-based activities with professional supervision demonstrated advantage in older adults, particularly in facilitating appropriate adaptations to perform different exercises or physical activities related to bones, muscles and cardiovascular system [22]. Group-based physical activities might result in other health benefits, including both physiological and social inclusion along with social support mechanisms involved in a group activity. Research suggested that club or team-based sports activities may improve mental health outcomes better than an individual workout program [23]. Another study suggested that the group exercise has great contribution to physical, psychological and social well-being of older adults. It also helped improve the functionality of health and social connections of older adults with their friends or peers and enjoy their lives [24]. While recognizing the benefits of group-based exercise intervention, a recent meta-analyses study identified big gaps in the previous exercise interventions for older adults, including a lack of studies investigating the benefits of group interventions, as well as the effect brought from professional supervision in the interventions [25]. In order to fill the gaps as aforementioned, the current study is to compare the effects of supervised group-based walking and non-supervised individual-based walking interventions on frailty, cognitive function and quality of life among inactive older adults in Saudi Arabia.

**Objectives**.

1. To compare the difference of effects between supervised group-based walking and non-supervised individual-based walking on the primary outcomes, including frailty, cognitive functions, and health-related quality of life among inactive older adults in Saudi Arabia.
2. To compare the difference of effects between supervised group-based walking and non-supervised individual-based walking on the secondary outcomes, including physical activity enjoyment, health parameters consisting of resting heart rate, resting blood pressure, and body composition, as well as walking performance among inactive older adults in Saudi Arabia.
3. To formulate guidelines for achieving an effective walking intervention for inactive older adults in Saudi Arabia.
4. To recommend the government of Saudi Arabia on guidelines and policies for promoting walking and active ageing among older adults in the country.

## Methods

### Study design

This study will apply a three-group (2 intervention groups and 1 control group), double-blinded (outcome assessors and data analysts), randomized controlled trial (RCT) to compare the effects of supervised group-based walking and non-supervised individual-based walking interventions on frailty, cognitive function and quality of life among older adults in the Kingdom of Saudi Arabia, immediately after a 16-week intervention, and 12-week residual effects after completion of the intervention. The double blinding adopted in this study is to reduce the bias and to ensure that those who are collecting the outcome measures (such as research assistants collecting data via questionnaires), and those who are inputting and analyzing the data, have no knowledge regarding group allocation status of the study participants. In assigning the groups, the CONSORT procedure will be followed [26]. The intervention groups will participate in a group-based walking intervention with professional trainer’s supervision and an individual-based walking intervention without professional trainer's supervision whereas the control group will not receive training but will be provided opportunity to receive the same training after the final assessment (12-week after completion of the intervention). This offer will be an incentive to the participants staying in the control group until the completion of the project. The study will be conducted following the international Good Clinical Practice Guidelines, by which the approval from the Research Ethics Committee of the Hong Kong Baptist University will be received prior to the commencement of the study.

### Design of intervention programs

Apart from the aforementioned effect of walking in nature on older adults’ physical and psychosocial well-being, the characteristics and style of the intervention were also seen as influencing factors in affecting the effectiveness of exercise intervention, including walking intervention. In recent older adults exercise interventions meta-analysis [25], it indicated that results from group exercise intervention studies were inconsistent, in which more studies investigating the differences between group and individual exercise interventions, the effects of professional delivery and significant others on exercise intervention outcomes were encouraged. Given that group interventions were most likely to be applied in psychological therapy, group exercise interventions refer to exercise classes that involve group-dynamics principles and could be used to increase participants’ cohesiveness and the effect of social support [27]. Moreover, group exercise interventions tended to be systematic and supervised by professionals. A meta-analysis study pointed out that group exercise intervention with professional supervision shown more superior results in functional effectiveness, adherence, quality of life and social interaction compared to that of individual-based intervention without professional supervision [27]. Therefore, in order to respond to the gap indicated in Di Lorito and his team’s meta-analysis work, the current intervention is proposing to conduct a randomized control trial that is not only examining the nature of walking on older adults’ well-being but also involving the elements of group-based and professional supervision intervention, aiming at comparing with the individual-based intervention without professional supervision. Furthermore, with the expected outcomes of the proposed study, new policy initiatives regarding the importance of having regular professional trainers on duty in public sport and exercise centers could be proposed, and yet help to extend the potential market of professional exercise trainers in Saudi Arabia and potentially in other countries.

### Participants

The participants will be recruited in the Kingdom of Saudi Arabia (KSA) using the convenience sampling. Considering KSA, the most physically inactive age group is 55–64 years [16], and based on United Nations the minimum age defined as older adult is 60 [1], this study will target the older adults, males and females, with age from 60–70. The eligibility criteria for selection of subjects include: (1) 60–70 years old; (2) capable of walking without assistive device; (3) healthy and live independently in communities; (4) no cardiovascular or related diseases that prevent from intensive walking; (5) pass the PAR-Q screening or with physician’s advice on readiness of participation in exercise training; (6) with no diagnosed cognitive impairment; and (7) being physically inactive. Physically inactive refers to older adults who have not met the PA recommendation stated by the World Health Organization. The PA recommendation refers to “Older adults should do at least 150 minutes of moderate-intensity aerobic physical activity throughout the week or do at least 75 minutes of vigorous-intensity aerobic physical activity throughout the week or an equivalent combination of moderate- and vigorous-intensity activity” adopted by WHO (2020).

All participants will be recruited following the principle of voluntariness and their data will be kept confidential. Informed consent letters will be signed by the participants prior to the commencement of the intervention. Participants who complete all three-time points of assessments and achieve 70% of attendance (the intervention group) will receive a grocery voucher value at $20 USD as a token of appreciation upon completion of the study.

### Sample size estimation

G*Power was used to determine the sample size. Based on the previous intervention studies, the most frequently reported effect size was ranged from moderate (0.25) to high (0.70) [28, 29], a moderate effect size of 0.25 will be adopted for the current study. To ensure a power of 80% probability for detecting a treatment difference at a two-sided 5% level of significance, a sample size of 35 participants per group (total 105 for 3 groups) is expected to recruit. Yet, considering a 20% of potential dropout rate, a sample size of 42 participants per group (total 126 for 3 groups) will be required in the current study for the two-way repeated measures in three-time points. It is decided a total of 126 participants will be recruited. In order to satisfy the culture of KSA that males and females should undergo walking intervention separately in the group, an equal number of males and females (63 M and 63 F) will be recruited and randomly and equally assigned to the two intervention groups and control group. Moreover, in order to have an approximate mean age in each intervention group, the participants will be divided into 2 groups (60–64 and 65 to 70) for the randomization.

### Grouping and randomization

**Fig. 1:**
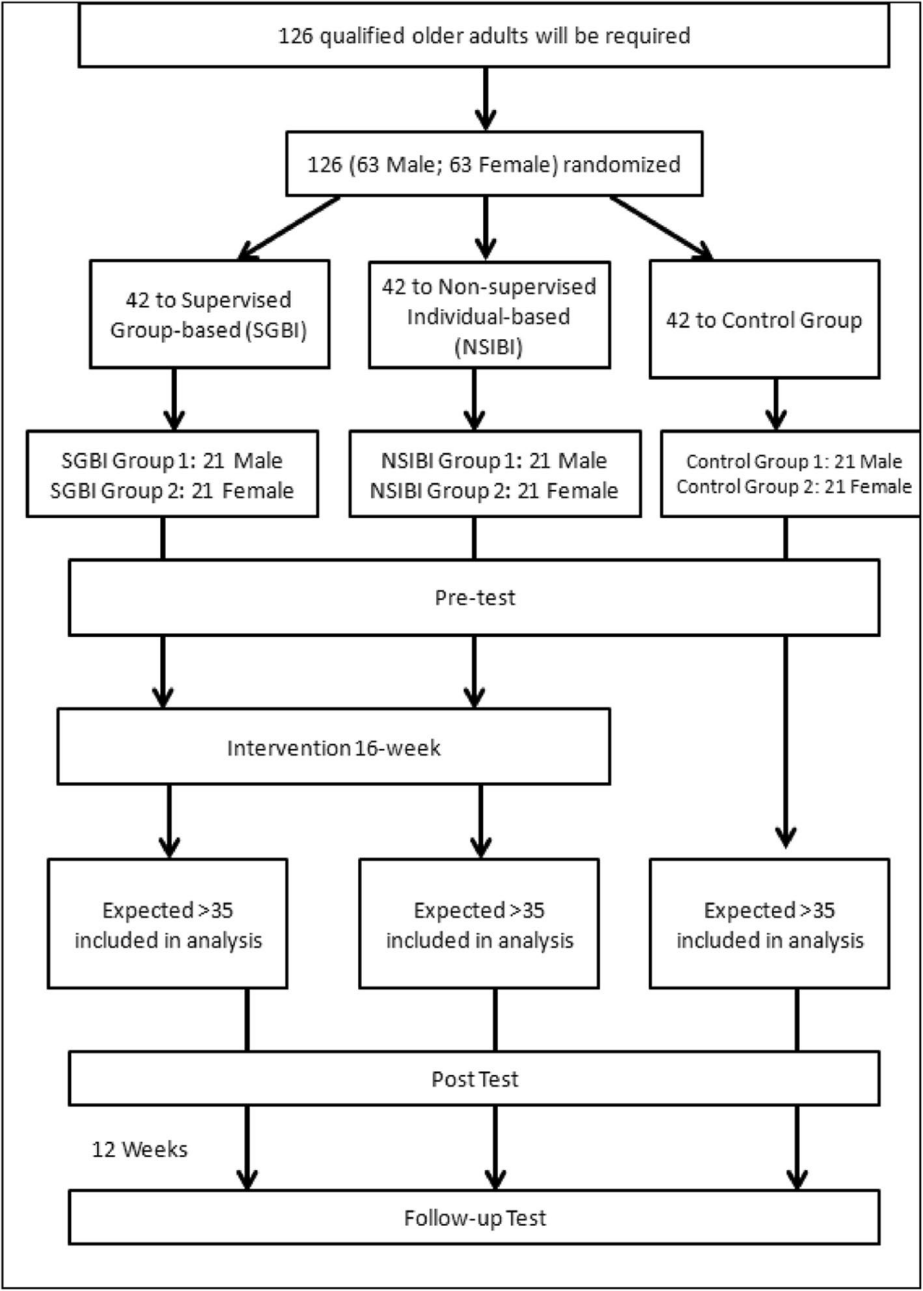
CONSORT Diagram for participants recruitment and intervention

Qualified male (*n* = 63) and female (*n* = 63) participants who have signed a consent letter will be randomly assigned into the three groups by a draw of lots, in a ratio of 1:1. The three groups will be (1) Supervised Group-based Intervention (SGBI; *n* = 42); (2) Non-supervised Individual-based Intervention (NSIBI; *n* = 42) and (3) Control Group (CG) (*n* = 42). The CONSORT procedure [26](Schulz et al., 2010) will be followed for the assignment and collection of data (Fig. 1).

### The intervention

#### Prescription of walking intervention

The walking intervention will be conducted in duration of 16 weeks, with 3 sessions per week and 50–70 min per session. As respects to the culture of KSA, male and female participants will undergo intervention in separate groups, with supervision or assistance by professional trainers or research assistants in the same gender during the intervention. In intervention program, the ACSM progressive training principle will be adopted. The intensity and repetition will be progressively increased in accordance with the 4 levels as prescribed, in which weeks 1 to 4 are Level-1; weeks 5 to 8 are Level-2, weeks 9 to 12 are Level-3, weeks 13–16 are Level-4 (Please refer to Table 1 for the detailed intervention program). The levels of progression could ensure the physically inactive participants have sufficient time to equip with an appropriate base of fitness and familiar with regular walking regime, as well as to prevent musculoskeletal injuries and over training. Also, the design of the training sessions will include the expected target heart rates, the intensity and the expected number of steps. Most importantly, each participant will be required to put on a wrist-worn Fitbit Charge 3 to keep track of their exercise intensity levels, including their heart rate changes in relation to their target heart rate zones, and the accumulated amount of steps after each session. In addition to progression, the training principle of individuality will also be applied to the participants doing the walking. They will be allowed to complete the targeted steps based on their own targeted heart rates as well as physical conditions during walking. Participants are free to taking short breaks or withdraw from walking if they uncomfortable doing it according to the schedule. Furthermore, in order to minimize the external influences caused by environmental settings, both intervention groups will receive the training at the same indoor track/sports paradise, with air-conditioning to produce a constant temperature.

**Table 1:** Intervention Protocol (Coswig et al., 2020 ; Harris et al., 2017; Haynes et al., 2020; Ignaszewski et al., 2017;Tudor-Locke et al., 2018)

Intervention Group	SGBI	NSIBI
Condition Warm-up (stretching): 5–10 min Walking: 20-40 min Cool-down (stretching): 5-10 min	Supervised group-based intervention Manpower: Research Assistant & Qualified Fitness Trainer Equipment: Fitbit Charge 3 Venue: Indoor Sport Paradise	Non-supervised individual-based intervention Manpower: Research Assistant Equipment: Fitbit Charge 3 Venue: Indoor Sport Paradise
**Level-1** **1–4 Weeks** **Low-to-Moderate Intensity**	**Duration**: 50 min. 3 times per week **Expected Intensity**: 50–60% of HRmax **Targeted steps**:3000/session **Parameters**: Heart rate Total number of steps	**Duration**: 50 min. 3 times per week **Expected Intensity**: 50–60% of HRmax **Targeted steps**:3000/session **Parameters**: Heart rate Total number of steps
**Level - 2** **5–8 Weeks** **Moderate Intensity**	**Duration**: 60 min. 3 times per week **Expected Intensity**: 50–60% of HRmax **Targeted steps**:4500/session **Parameters**: Heart rate Total number of steps	**Duration**: 60 min. 3 times per week **Expected Intensity**: 50–60% of HRmax **Targeted steps**:4500/session **Parameters**: Heart rate Total number of steps
**Level-3** **9-12 Weeks** **Moderate-to-high Intensity**	**Duration**: 60 min. 3 times per week **Expected Intensity**: 55–65% of HRmax **Targeted steps**:7000/session **Parameters**: Heart rate Total number of steps	**Duration**: 60 min. 3 times per week **Expected Intensity**: 55–65% of HRmax **Targeted steps**:7000/session **Parameters**: Heart rate Total number of steps
**Level-4** **13–16 Weeks** **Moderate-to-vigorous Intensity**	**Duration**: 70 min. 3 times per week **Expected Intensity**: 65–80% of HRmax **Targeted steps**:8500–10,000/session **Parameters**: Heart rate Total number of steps	**Duration**: 70 min. 3 times per week **Expected Intensity**: 65–80% of HRmax **Targeted steps**:8500–10,000/session **Parameters**: Heart rate Total number of steps

Additionally, to respect the culture of KSA, male and female participants will be separated to undergo the walking intervention. In such arrangement, there will be 4 groups [2 groups (1 male and 1 female) under group-based and 2 groups (1 male and 1 female) under individual-based] and each group will have 21 participants when doing walking. While qualified professional fitness trainers (1 male and 1 female separately for male and female groups, respectively) will be involved in the group-based intervention programs in order to provide appropriate guidance and supervision to the participants. Whereas, the non-supervised individual-based intervention will be conducted by a research assistant (males and females) to ensure the attendance and general safety, but without providing professional advice or supervision.

Upon completion of the 16-week walking interventions, only data of the participants who have attended at least 70% of the training sessions will be included in the analyses.

### The control group

Participants in the control group (CG) (21 males and 21 females) will not participate in any specific intervention during the whole study period (the 16-week intervention and 12-week follow-up periods), but they will be asked to keep a daily log of their physical activity, use of medicines, illness, and other health-related activities. In addition, the CG participants will be asked to report to the research assistant (RA) if a major change has been made in the aforementioned aspects. The RA will also check the daily logs of the participants through a telephone or mobile phone every two weeks. Data from those who had changed their normal lifestyles (especially taking up regular physical activity) will be examined for exclusion in the subsequent data analysis.

### Outcome measures

#### Primary outcomes

##### Health-related quality of life

The Short Form-36 (SF-36) is a widely used health survey questionnaire, especially for older adults to assess the health-related quality of life (HRQoL) [30]. The SF-36 consists of 36 items covering two dimensions—physical health (physical functioning, role limitations due to physical problems, role limitations due to emotional problems, and social functioning) and mental health (mental health, vitality, body pain, and general health perception). In the current study, the SF-36 (SF-36) will be used [31].

### Frailty

Physical Performance Test (PPT) is one of the tools which are used to assess the level of frailty [32]. It is usually used in several domains of physical function through the observed performance of various tasks that are important to simulate the activities of daily living of various degrees of complexity. It is a very simple test that usually requires about 10 min. It is developed in two versions including 9 and 7 item scale. In our research we will use the 9 item scale that includes 9 standardized tasks such as writing a sentence, simulated eating, turning 360 degrees, putting on and removing a jacket, lifting a book and putting it on a shelf, picking up a penny from the floor, a 50-foot walk test, and climbing stairs (scored as two items). The score range of each task is 0–4 and for 9 items it will be 36. A higher score indicates better performance [32].

### Cognitive function

The Mini-mental state examination (MMSE) is the most commonly used method to measure the level of cognitive impairment, especially in older adults. It is also regarded as an effective instrument to screen the severity of cognitive impairment at a given point in time and also to observe the cognitive changes in a person over time as well as to get information about the individual’s response to the particular treatment option [33]. It is a 10–15-minute test procedure that involves several sub-division of cognitive status, which includes memory, language, attention, orientation and also visuospatial proficiency. The score of Mini-mental state examination is calculated on a scale of 0–30 where score 24 or above is usually considered as the normal cognitive status or no cognitive impairment of the individual. The overall scorings are Severe cognitive impairment (0–17), Mild cognitive impairment (18–23) and no cognitive impairment (24–30).

### Secondary outcomes

#### *Health parameters* - *Body composition, resting heart rate & resting blood pressure*

The walking effects on participants’ health parameters, namely body composition, resting heart rate, and resting blood pressure will also be assessed. The body composition will be assessed by Tanita MC780U (Tanita Corporation, 2020), by which the percent body fat and lean body mass of participants will be recorded. The body mass index (BMI) using participants’ heights and weights (measured by the RA) will also be recorded. The wrist-worn Fitbit Charge 3 will be used to record the resting heart rate of participants (also the heart rates during walking in reflecting the exercise intensity of the participants as well as the accumulated footsteps and the duration of the completed footsteps during each session of the intervention for performance analyses). The resting blood pressure of the participants will be recorded by Lenus Automatic Blood Pressure Monitor DP65. All the assessments will be conducted in the pre-test, post-test and follow-up test for comparisons.

### Physical activity enjoyment

The 8-item Physical Activity Enjoyment Scale (PACES)[34] will be applied to measure the participants’ level of enjoyment after engaging in walking intervention programs. The 8-item Physical Activity Enjoyment Scale is rated by a 7 bipolar rating, with items such as " I enjoy it.“ (One extreme) to " I dislike it.“(the other extreme). In the scale, the negative items (i.e., 1, 4, 5, and 7) requires reverse coding, and the Higher PACES score the greater levels of physical activity enjoyment demonstrated. The 8-item PACES showed excellent internal consistency and reliability among the older adult population. Furthermore, the scale showed adequate validity and show no measurement invariance between genders.

#### Walking performance

The time for completing the targeted steps in targeted heart rate zones of each participant in each training session will be recorded for analysis of performance and improvement on walking. All the data will be recorded by the wrist-worn Fitbit Charge 3 and retrieve from the computer after each training session. .

### Intervention experience evaluation

To further investigate and compare the perception of the participants after the intervention, a few open-ended questions regarding their feelings and attitude towards the intervention, as well as their physical activity behavior after the intervention will be asked in the follow-up test. (Please refer to Appendix for the examples of the questionnaire items)

### Statistical analysis

Descriptive statistics will be used to describe baseline characteristics such as gender, age, living status (alone or with family), body height, body weight, resting heart rate, and blood pressure of the participants in the study. The primary outcomes will be analyzed using an intention-to-treat (ITT) approach, and a sensitivity test will subsequently be conducted using the available data. Missing data will be replaced using the last observation carried forward method. For the secondary outcomes, a per-protocol analysis will be conducted using the available data. The statistical significance level was set at *p* < .05. All data will be analyzed using SPSS Version 26.0 (IBM, Chicago, IL). For the within-group test, a one-way repeated measure analysis of variance (ANOVA) will be applied to each group to determine the changes of each outcome parameter among the three-time points (i.e., pretest, posttest, and follow-up test). When the sphericity assumption was violated, the Greenhouse– Geisser correction will be used when epsilon < 0.75, or the Huynh–Feldt correction will be used when epsilon > 0.75. Post hoc analysis with Bonferroni correction will be conducted to explore the differences between the pretest and posttest, as well as posttest and follow-up test. The mean differences with 95% confidence intervals (CIs) will be reported. For the inter-group test, a one-way analysis of covariance (ANCOVA) with the baseline value as a covariate will be conducted at the posttest, and follow-up test to determine the group effect on each outcome parameter. Subsequently, the differences between the intervention groups and the control group will be analyzed through planned contrasts.

#### Discussion and conclusion

The purpose of the proposed study is to compare the effects of supervised group-based walking and non-supervised individual-based walking interventions on frailty, cognitive function and quality of life among inactive older adults in Saudi Arabia. We are aware that walking related research in Saudi Arabia has tended to be cross-sectional and environment based. Using questionnaire surveys and GIS applications, environmental researchers determined that 80% of participants expressed a liking for walking due to the average accessibility of walking routes. Additionally, the study revealed that improving sidewalk walkability should also increase pedestrian behavior. However, as Saudi Arabia is in arid regions, the hot weather could have decreased the motivation to walk. Therefore, it could be considered as a potential issue in the current study [20, 21]. Additionally, researchers found that older adults with chronic disease in Saudi Arabia spent less time on walking-related activities, for which more attention was required. While physical therapists have also used walking-related therapeutic techniques with stroke patients [35, 36]. Nevertheless, the findings of this study will not only help identify the difference in effects caused by supervised group-based walking and non-supervised individual-based walking interventions, but they will also create awareness in Saudi Arabia among government and the general public about the ageing problem and ways to improve intervention outcomes. With the evidence, the government, universities, and the related non-governmental organizations in Saudi Arabia will have strong justifications for formulating guidelines and policies, and more importantly allocating more resources to promoting healthy and active ageing in their country.

## Data Availability

Not Applicable.

## Abbreviations

WHO: World Health Organization
KSA: Kingdom of Saudi Arabia
PACES: Physical Activity Enjoyment Scale
HRQoL: Health-related quality of life
PPT: Physical Performance Test
MMSE: The Mini-mental state examination
ANOVA: One-way repeated measure analysis of variance
ANCOVA: One-way analysis of covariance
